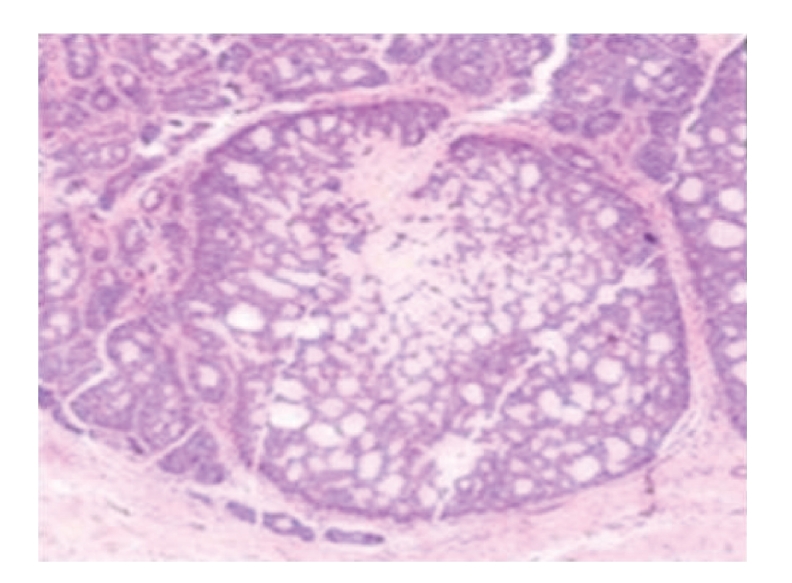# Dermoscopy of Cylindroma

**DOI:** 10.1155/2010/285392

**Published:** 2010-08-24

**Authors:** Horacio Cabo, Florencia Pedrini, Emilia Cohen Sabban

**Affiliations:** ^1^Department of Dermatology, Roffo Hospital, Avenue San Martín 5481, Villa del Parque, Buenos Aires C1417DTB, Argentina; ^2^Department of Dermatology, CEMIC, Talcahuano 1234, Buenos Aires C1014ADB, Argentina; ^3^Department of Dermatology, Tornu Hospital, Donato Alvarez 3002, Villa Ortuzar, Buenos Aires C1427ARN, Argentina

## Abstract

*Background*. Dermoscopy provides additional criteria for the diagnosis of nonpigmented skin lesions. *Case Report*. An 80-year-old woman presented with an isolated, firm, dome-shaped, erythematous nodule with surface telangiectasia on her forehead. Dermoscopy showed areas of background pink coloration with arborizing telangiectasia, blue dots/globules, and ulceration. Histologic analysis revealed features of cylindroma. *Conclusion*. Our case suggests that cylindromas may be added to the list of adnexal tumors mimicking BCC.

## 1. Introduction

Cylindromas are benign adnexal proliferations. They can present singly or in grouped numbers [1]. Single lesions commonly involve the head and the neck, especially the scalp. They can also develop on the skin of the trunk or genitalia [1]. 

Dermoscopy is a noninvasive technique which has greatly improved the diagnostic accuracy of pigmented skin tumors. And it can also be applied to nonpigmented skin lesions, such as adnexal tumors.

## 2. Case Report

An 80-year-old woman presented with an isolated, firm, dome-shaped, erythematous nodule with surface telangiectasia on her forehead. The size was 4 mm. She reported that the lesion appeared 10 months ago. 

**Figure 1:**
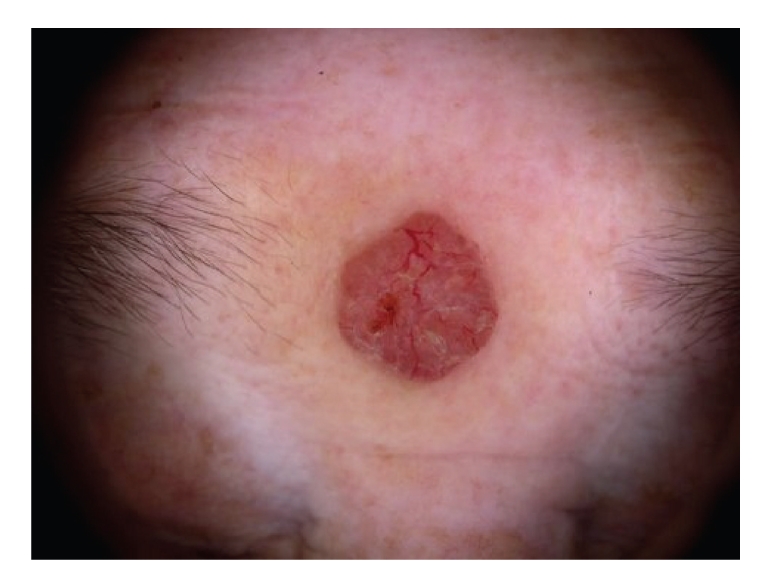
Polarized light dermoscopy.

**Figure 2:**
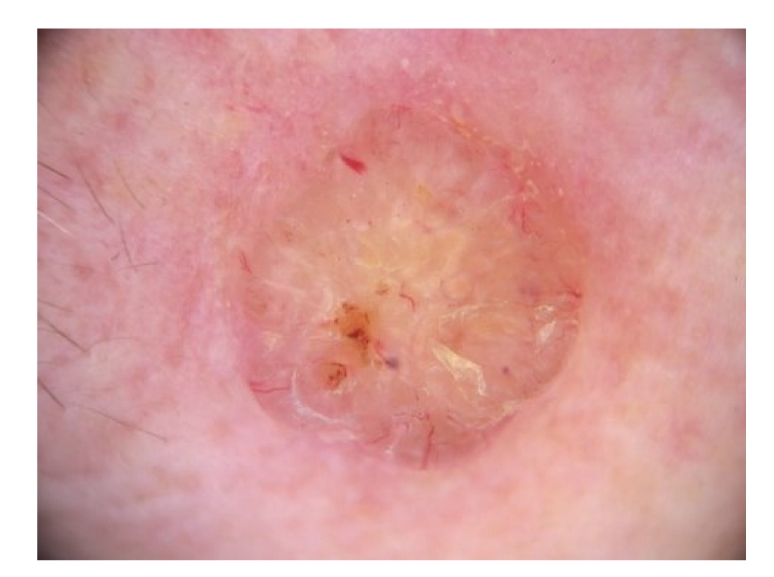
Contact dermoscopy.

Polarized light dermoscopy showed areas of background pink coloration with arborizing telangiectasia and ulceration (Figure 1). Contact dermoscopy showed a yellowish nonhomogenous area correlating to hyperkeratosis. Additionally few blue dots/globules were seen. (Figure 2). 

The nodule was excised. 

Histological sections stained by hematoxylin-eosin revealed nest of basaloid cells in a jigsaw puzzle-like pattern. Many nests were surrounded by a dense eosinophilic basement membrane material (Figure 3).

## 3. Discussion

Cylindromas are undifferential or poorly differentiated adnexal neoplasm of apocrine lineage. Cylindromas can be singly or in grouped numbers, and they are not clinically distinctive. A biopsy specimen is required for diagnosis [1]. Scalp cylindromas can become numerous, and may eventually cover the entire scalp, resulting in the so-called turban tumors. Partial or complete hair loss may be an associated finding [2]. The recognition of multiple cylindromas should prompt consideration of the Brooke-Spiegler syndrome, an autosomal dominant condition, some examples of which are associated with the CYLD gene on the chromosome 16q [1]. Although cylindromas are usually benign neoplasms, malignant transformation to cylindrocarcinomas is rare but well documented. Malignant cylindromas may be locally aggressive, often metastasizes, and require careful followup surveillance [2, 3]. 

Dermoscopy showed areas of pink background coloration, arborizing telangiectasia, blue dots/globules, and ulceration [4]. Similar patterns have been reported for basal cell carcinoma, including arborizing telangiectasia, ulceration, and multiple blue/gray globules [5]. The only difference between BCC and cylindroma dermoscopy is the colour of the dots/globules. They are blue but not gray in cylindroma dermoscopy. 

*Histopathology*. At low magnification, cylindroma consists of sharply circumscribed nodules arrayed within the dermis, with frequent extension into the underlying subcutis. The nodules are composed of nests of basaloid cells in close apposition, arrayed in complex pattern that has been likened to a jigsaw puzzle. A rim of densely eosinophilic, PAS-positive basement membrane material commonly surrounds the individual nest, and “droplets” of similar composition are often scattered in the centers of the small nests [1]. 

Treatment methods for cylindromas include excision and Mohs micrographic surgery [6]. In addition, successful treatment with lasers such as the argon, CO_2_, and erbium:Yag plus CO_2_ have been reported in adnexal tumors of Brooke-Spiegler syndrome [7–9]. Medical treatments for cylindromas that are currently being tested include sodium salicylate and prostaglandin A1, which are thought to restore growth control by inhibiting NF-B activity [2, 10].

## 4. Conclusion

Our case suggests that cylindromas may be added to the list of adnexal tumors mimicking BCC [11]. More cases are necessary for establishing the characteristic features of cylindromas.

## Figures and Tables

**Figure 3:**